# 

**Published:** 1877-06

**Authors:** 


					﻿Vol. 24.
JUNE, 1877.
No. 6^
TWENTY-FOURTH YEAR.
TIALTJS
Journal of Health.
PUBLISHED AT BIBLE HOUSE,
THIRD AVENUE, CORNER OF NINTH STREET,
INTE'W YORK.
E. H. GIBBS, M.D., Editor.
AGENTS m THE TRADE:
AMERICAN NEWS COMPANY, 121 NASSAU STREET.
NEW YORK NEWS COMPANY, 18 BEEKMAN STREET.
Terms, $ 1.50 a Year.
SINGLE NUMBER, 15 CENTS.
Kent & Co.. Printers, 11 Frankfort St., New Teak.
				

